# ADAMTS4 is involved in the production of the Alzheimer disease amyloid biomarker APP669-711

**DOI:** 10.1038/s41380-023-01946-y

**Published:** 2023-02-01

**Authors:** Masaya Matsuzaki, Miyabishara Yokoyama, Yota Yoshizawa, Naoki Kaneko, Hiroki Naito, Honoka Kobayashi, Akihito Korenaga, Sadanori Sekiya, Kentaro Ikemura, Gabriel Opoku, Satoshi Hirohata, Shinichi Iwamoto, Koichi Tanaka, Taisuke Tomita

**Affiliations:** 1 Laboratory of Neuropathology and Neuroscience, Graduate School of Pharmaceutical Sciences, The University of Tokyo, Tokyo, 113-0033 Japan; 2 Koichi Tanaka Mass Spectrometry Research Laboratory, Shimadzu Corporation, Kyoto, 604-8511 Japan; 3 Department of Medical Technology, Graduate School of Health Sciences, Okayama University, Okayama, 700-8558 Japan

**Keywords:** Neuroscience, Biochemistry

## Abstract

Amyloid-β (Aβ) deposition in the brain parenchyma is one of the pathological hallmarks of Alzheimer disease (AD). We have previously identified amyloid precursor protein (APP)669-711 (a.k.a. Aβ(-3)-40) in human plasma using immunoprecipitation combined with matrix-assisted laser desorption ionization time-of-flight mass spectrometry (IP-MALDI-MS). Furthermore, we found that the level of a composite biomarker, i.e., a combination of APP669-711/Aβ1-42 ratio and Aβ1-40/Aβ1-42 ratio in human plasma, correlates with the amyloid PET status of AD patients. However, the production mechanism of APP669-711 has remained unclear. Using in vitro and in vivo assays, we identified A Disintegrin and Metalloproteinase with a Thrombospondin type 1 motif, type 4 (ADAMTS4) as a responsible enzyme for APP669-711 production. ADAMTS4 cleaves APP directly to generate the C-terminal stub c102, which is subsequently proteolyzed by γ-secretase to release APP669-711. Genetic knockout of *ADAMTS4* reduced the production of endogenous APP669-711 by 30% to 40% in cultured cells as well as mouse plasma, irrespectively of Aβ levels. Finally, we found that the endogenous murine APP669-711/Aβ1-42 ratio was increased in aged AD model mice, which shows Aβ deposition as observed in human patients. These data suggest that ADAMTS4 is involved in the production of APP669-711, and a plasma biomarker determined by IP-MALDI-MS can be used to estimate the level of Aβ deposition in the brain of mouse models.

## Introduction

Alzheimer disease (AD) in particular accounts for a large population of the causes of dementia [1]. Senile plaques, which are formed by the aggregation and deposition of amyloid-β (Aβ), and neurofibrillary tangles composed of tau protein, are the pathological features observed in the brains of patients with AD. Aβ is produced by the proteolytic cleavage of amyloid precursor protein (APP) by β- and γ-secretases [1–3]. Several genetic mutations linked to familial AD increase the production or aggregation of Aβ. In contrast, patients with sporadic AD have been reported to have a decreased clearance rate of brain Aβ, rather than its increased production rate [4]. These data strongly indicate that the accumulation of Aβ triggers the onset of AD. However, the deposition of Aβ in the brain starts 10–20 years before the appearance of the clinical symptoms of AD. Moreover, recent results of clinical trials have implicated that anti-Aβ treatment should be performed on patients in the mild cognitive impairment or prodromal AD stage for beneficial effects [5]. However, cognitive function tests such as Mini-Mental State Examination (MMSE) and Clinical Dementia Rating (CDR) are difficult to diagnose the amyloid deposition in the asymptomatic stage. Amyloid imaging using positron emission tomography (PET) and quantification of Aβ42 and tau levels in the cerebrospinal fluid (CSF) is utilized to estimate the brain Aβ deposition. However, it is difficult to identify amyloid deposition in subjects in the asymptomatic stage using cognitive function tests, such as MMSE and CDR. Amyloid imaging using PET, and the quantification of Aβ42 and tau levels in the CSF are utilized to estimate brain Aβ deposition levels. However, these methods are expensive or highly invasive. Thus, determination of the pathological stage of brain amyloid deposition, and estimation of AD risk by a non-invasive and cost-effective method is essential for the development of anti-Aβ therapeutics.

The N- and C-terminal lengths of Aβ are heterogeneous. The dominant Aβ species in senile plaques start with a pyroglutamate residue at the 3rd position and end with an alanine at the 42nd position [6–8]. However, the major secreted form of Aβ in the conditioned medium of cultured cells is Aβ1-40 [9, 10]. These heterogeneities are due to proteolytic modifications after Aβ deposition and multiple cleavage mechanisms during the Aβ-generating process [2]. In particular, γ-secretase cleaves APP at multiple sites, resulting in the generation of Aβ ending at the 37th, 38th, 40th, 42nd, and 43rd positions. For the N-terminal side, the β-secretase BACE1 cleaves Aβ mainly at the 1st and 11th positions. In addition, other enzymes have been implicated in the generation of N-terminally truncated Aβ species, which have been identified from the conditioned medium of cultured cells. However, N-terminally elongated Aβ species have not been investigated to date. In recent years, we identified a novel N-terminally elongated Aβ, APP669-711 (a.k.a. Aβ(-3)-40), in human plasma using an ultrasensitive immunoprecipitation (IP) method combined with matrix-assisted laser desorption ionization time-of-flight mass spectrometry (IP-MALDI-MS) [11]. Importantly, the plasma APP669-711/Aβ1-42 ratio is increased in amyloid PET-positive AD patients. Furthermore, a composite plasma biomarker combining APP669-711/Aβ1-42 ratio and plasma Aβ1-40/Aβ1-42 ratio was shown to enable the estimation of brain Aβ accumulation in amyloid PET-positive individuals with 90% accuracy using 0.5 ml of blood [12]. Its sensitivity and specificity were 91.7% to 100% and 84.5%, respectively, indicating a separability comparable to that of amyloid PET. Head-to-head comparison analysis demonstrated that this composite biomarker showed the highest correlation with CSF Aβ42/40 ratio [13]. However, how APP669-711 is produced, and the mechanisms that regulate APP669-711 levels remain unclear. In this study, we found that the secreted metalloprotease, a disintegrin and metalloproteinase with thrombospondin motifs 4 (ADAMTS4), and γ-secretase are involved in the generation of APP669-711, both in vitro and in vivo.

## Materials and methods

### Animals

All experiments using animals were performed according to the guidelines provided by the Institutional Animal Care Committee of Graduate School of Pharmaceutical Sciences, The University of Tokyo (Protocol no. P29-30 and P30-3), and Graduate School of Health Sciences, Okayama University (#OKU2022-413). APP/PS1 mice (B6.Cg-Tg(APPswe, PSEN1dE9)85Dbo/Mmjax, Jackson Laboratory, JAX mouse #005864) and *Adamts4*^*−/−*^ mice (B6.129P2-Adamts4tm1Dgen/J, Jackson Laboratory, JAX mouse #005770) were used. Detailed experimental methods are described in the Supplementary Information.

### Antibodies, chemicals, plasmids, cells, and immunological methods

For the generation of CRISPR knockout cells, we used Cas9 nickase (D10A) which requires two adjacent guide RNAs for cleaving target regions [14–16]. cDNAs encoding gRNA sequences (Supplementary Information) were inserted into pX335-U6-Chimeric_BB-CBh-hSpCas9n(D10A) (a gift from Dr. Feng Zhang, Addgene plasmid #42335; http://n2t.net/addgene:42335; RRID: Addgene_42335) and pBabe Puro U6 BbsI (kindly provided by Dr. Dario Alessi (University of Dundee). Transfection into HEK293A and A549 cells using polyethylenimine (Polysciences) and lipofectAMINE LTX (Invitrogen), respectively, were described previously [14, 15]. A549 cells have 5.8 ± 1.40 copies of chromosome 1 per karyotype [17], in which the *ADAMTS4* gene locates. Thus, we had chosen the monoclonal cell line that harbors three mutations within the target genomic sequence. Materials, detailed protocol for cultured cells, molecular biology, and immunological methods are described in the Supplementary Information.

### In vitro cleavage assay

Plasmids encoding APP81 are transformed into *E. coli* BL21(DE3) (Novagen), and the substrate were purified as described in the Supplementary Information. Same volume (80 μl) of 17 μM APP81 and 100 nM ADAMTS4 protein (diluted in assay buffer (50 mM HEPES, 50 mM NaCl, 1 mM CaCl2, 0.05% Brij-35, pH 7.5)) were mixed and incubated at 37 °C for indicated hours. For immunoblotting, the reaction was halted by the addition of 2x Laemmli sample buffer. For MALDI-TOF MS, the mixture was stored at −80 °C until use.

### MALDI-TOF MS

Mass spectra were acquired using a MALDI-linear TOF mass spectrometer (AXIMA Performance, Shimadzu/KRATOS, Manchester, UK) in the positive ion mode. MS/MS analysis for the identification of the proteolyzed peptides was performed using a MALDI-QIT reflectron TOF mass spectrometer (AXIMA Resonance, Shimadzu/KRATOS, Manchester, UK) in the positive ion mode and MS/MS product ions were generated by collision-induced dissociation with argon gas. The *m/z* reported in the linear TOF and the QIT-reflectron TOF represent the average and monoisotopic peak of the protonated signal [M + H]^+^, respectively. The *m/z* value was calibrated with human angiotensin II, human ACTH fragment 18-39, bovine insulin oxidized beta-chain, and bovine insulin. Detailed protocol for solid-phase extraction, IP-MALDI-MS, and MS/MS analyses are described in the Supplementary Information.

### Statistical analysis

All samples were analyzed in a randomized manner. For quantitative analyses, Student’s *t* test was used for comparisons between two-group data, and Tukey’s test was used for multiple group comparisons. Statistical analyses were performed by KyPlot or Excel software. In the figures, statistical results were indicated by absolute *p* values. A *p* value <0.05 was considered to have a significant difference.

Experimental details are described in the Supplementary Information.

## Results

### APP669-711 is proteolytically generated from various cells under physiological conditions

**Fig. 1:**
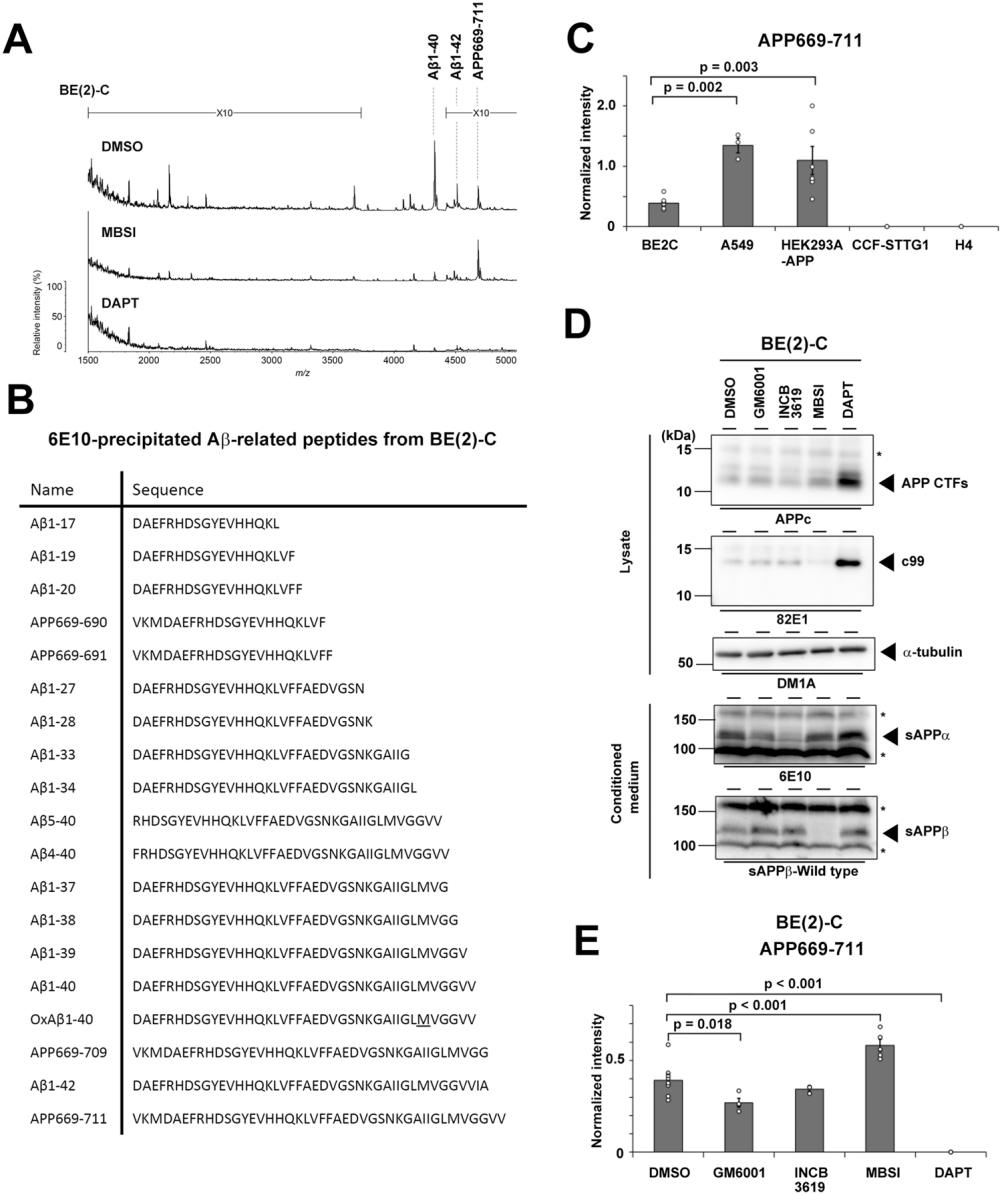
Detection of APP669-711 in the conditioned medium of cultured cell lines. **A** IP-MALD-MS spectrum of the 6E10 antibody-precipitated endogenous Aβ peptide variants in the conditioned medium from BE(2)-C cells. Various Aβ peptides, which are diminished by either MBSI or DAPT treatment, were identified. **B** The identified Aβ peptides secreted in **A**. OxAβ1-40 contained an oxidized methionine at the Aβ35th residue. **C** Comparison of the level of endogenous APP669-711 in the conditioned medium of human-derived cultured cells (*n* = 4–6, mean ± s.e.m. Tukey test). **D** Pharmacological effects of secretase inhibitors on APP metabolism in BE(2)-C cells. Asterisks indicate non-specific bands. **E** Pharmacological effects of secretase inhibitors on the production of APP669-711 from BE(2)-C cells (*n* = 4 or 5, mean ± s.e.m. Tukey test).

Although γ-secretase-mediated C-terminal variations of Aβ have been extensively analyzed, the N-terminal variations of secreted Aβ have not been investigated to date. Importantly, several analyses of cell-based models and genetically modified animals harboring the APP mutant carrying the Swedish mutation (APPswe) located at the (−1st) and (−2nd) positions of Aβ, which significantly increases the β-secretase-mediated cleavage at the 1st position, to induce the overproduction of Aβ have been performed [18, 19]. However, the profiles of secreted Aβ from endogenous APP and overexpressed APPswe are distinct in mouse neuroblastoma Neuro2a cells [20]. We previously demonstrated using IP-MALDI-MS and the 6E10 antibody that human neuroblastoma BE(2)-C cells secrete endogenous APP669-711 as well as Aβ [12]. We further analyzed the conditioned media from several human-derived cell lines. We found that APP669-711 was secreted from A549 adenocarcinoma cells as well as HEK293A cells expressing wild-type APP (APPwt), but not CCF-STTG1 astrocytoma, H4 neuroglioma, nor naïve HEK293A cells (Fig. 1C), suggesting that APP669-711 is produced from only some cultured cell lines under physiological conditions. To investigate the mechanism of the generation of APP669-711, we analyzed the effects of several inhibitors that affect APP metabolism in BE(2)-C cells (Fig. 1D, E). The γ-secretase inhibitor DAPT abolished the production of APP669-711. In contrast, treatment with the specific and potent BACE1 inhibitor MBSI increased APP669-711 production. Notably, GM6001, a pan metalloprotease inhibitor, partially inhibited APP669-711 production, although the ADAM10/17-specific inhibitor INCB3619 did not affect APP669-711 levels. These data suggested that an unknown metalloprotease-mediated cleavage at the APP669 site (a.k.a. (−3rd) position in Aβ) is involved in the generation of APP669-711.

**Fig. 2:**
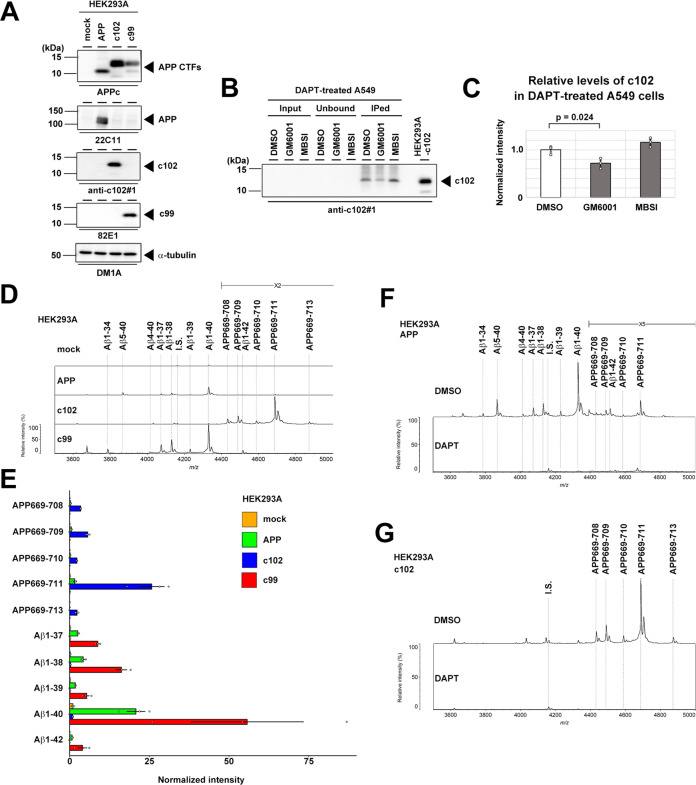
Processing of c102, a direct substrate for APP669-711. **A** Immunoblot analysis of HEK293A cells expressing APP, c102, and c99. Note that the N-terminal end-specific antibodies anti-c102 and 82E1 detected c102 and c99, respectively. **B** Detection of endogenous c102 in the lysate of A549 cells by IP using an anti-c102 antibody. Cells were treated with DAPT to increase c102 levels. **C** Changes in c102 levels by GM6001 or MBSI in A549 cells co-treated with DAPT (*n* = 3, mean ± s.e.m. Tukey test). **D** IP-MALD-MS spectrum of the 6E10 antibody-precipitated Aβ peptides in the conditioned medium from HEK293 cells expressing APP, c102, or c99. I.S. internal standard. **E** Levels of Aβ peptides secreted from HEK293A cells transfected with APP, c102, or c99 (*n* = 4 or 5 or 4, mean ± s.e.m.). **F** Effects of DAPT on the production of Aβ peptides of APP-expressing HEK293A cells. **G** Effects of DAPT on the production of Aβ peptides of c102-expressing HEK293A cells.

In the Aβ production pathway, APP is cleaved by BACE1 at the 1st position of Aβ to generate c99, which is a direct substrate for γ-secretase. According to the proteolytic model above, the APP669-site cleavage of APP results in the production of the C-terminal stub c102, which is c99 with 3 extra N-terminal amino acid residues. To detect endogenous c102, we generated N-terminal end-specific anti-c102 antibodies (anti-c102#1 for immunoblotting, and anti-c102#3 for immunohistochemistry). The specificity of the antibodies was confirmed by immunoblotting of HEK293A cell lysates expressing recombinant c99 or c102, the latter being a sequence with 3 extra amino acid residues between the signal peptide and Aβ sequence in the c99 expression vector (Fig. 2A). By IP using the anti-c102 antibody, we confirmed the appearance of endogenous c102 in the lysates of A549 cells upon DAPT treatment (Fig. 2B, C). Furthermore, cotreatment of GM6001 reduced c102 levels in A549 cell lysates, supporting our notion that APP669-site cleavage of APP is mediated by an unknown metalloprotease. To further confirm that c102 is a direct substrate for APP669-711, we analyzed the conditioned medium of HEK293A cells expressing APPwt, c99, and c102 by IP-MALDI-MS (Fig. 2D, E). As expected, we detected the Aβ1-37, Aβ1-38, Aβ1-39, Aβ1-40, Aβ1-42_,_ and APP669-711 (Aβ(-3)-40) in the media of APPwt-expressing cells. From recombinant c99, various Aβ species starting at the 1st position were identified. In contrast, the conditioned medium of cells expressing c102 contained various Aβ species starting at APP669 position (i.e., APP669-708 (Aβ(-3)-37), APP669-709 (Aβ(-3)-38), APP669-710 (Aβ(-3)-39), APP669-711 (Aβ(-3)-40), APP669-713 (Aβ(-3)-42)). DAPT treatment completely abolished the production of all Aβ species derived from APPwt and c102 (Fig. 2F, G). These data strongly indicate that APP669-711 is also proteolytically produced from APP by sequential cleavage by a yet-unknown metalloprotease at the APP669 site, and γ-secretase within the transmembrane domain.

**Fig. 3:**
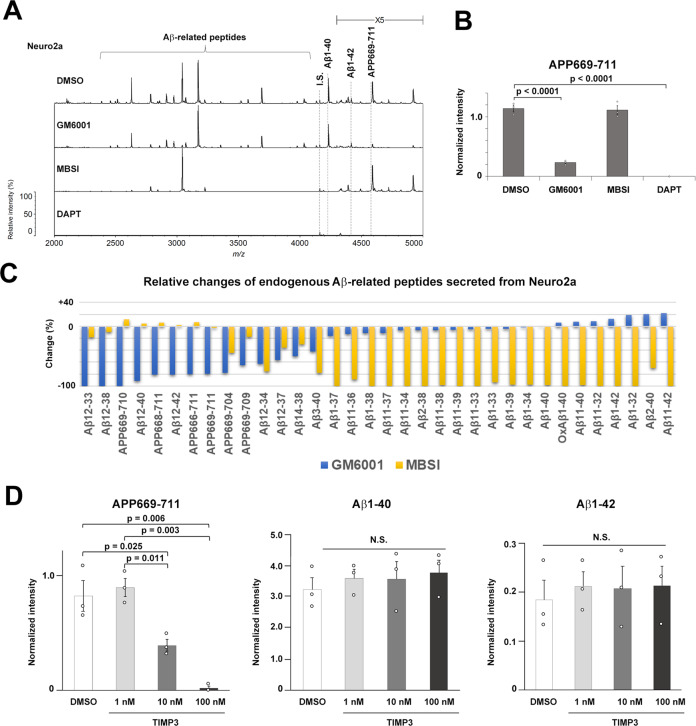
Metalloprotease-dependent production of APP669-711 from Neuro2a cells. **A** IP-MALD-MS spectrum of the 4G8 antibody-precipitated endogenous Aβ peptides in the conditioned medium from Neuro2a cells. Various Aβ peptides were identified. I.S. internal standard. **B** The pharmacological effect of secretase inhibitors on the production of APP669-711 from Neuro2a cells (*n* = 3, mean ± s.e.m. Tukey test). **C** Comparison of the effects of GM6001 and MBSI on the production of endogenous Aβ peptides in Neuro2a cells. **D** Effects of TIMP3 treatment of on the APP669-711 and Aβ production from Neuro2a cells (*n* = 3, mean ± s.e.m. Tukey test. N.S. not significant).

We then comprehensively analyzed the endogenous Aβ species secreted from mouse neuroblastoma Neuro2a cells by IP-MALDI-MS. For IP, instead of the 6E10 antibody, which is a human Aβ-specific antibody, we utilized the 4G8 antibody targeting the common sequence in the middle of human and murine Aβ (Fig. 3A, B). We successfully detected 34 types of Aβ-associated peptides in the cultured medium (Fig. 3C). As observed in human-derived cultured cells, GM6001 treatment of neuro2A cells significantly reduced the production of endogenous APP669-711, whereas MBSI did not affect APP669-711 levels. Intriguingly, treatment with MBSI, but not GM6001, abolished the production of Aβ species starting at the 1st, 2nd, and 11th positions, suggesting that BACE1 activity is specifically required for the proteolysis of APP at these sites. In contrast, the production of Aβ species starting at the APP669 and Aβ 12th positions was significantly reduced by GM6001, but not MBSI. Furthermore, we analyzed the effects of the Tissue inhibitor of metalloproteinase 3 (TIMP3), which is an endogenous potent metalloprotease inhibitor implicated in AD pathogenesis [21, 22], on secreted APP669-711 levels from Neuro2a cells (Fig. 3D). Notably, TIMP3 treatment completely inhibited the production of endogenous APP669-711, whereas secreted Aβ levels were unaltered. A comparison of absolute levels of Aβ1-40 and APP669-711 indicated that the processing ratio of BACE1 and ADAMTS4 on APP is 1:0.15. We then estimated the proportion of APP undergoing processing by BACE1 and α-secretase by end-specific ELISAs for sAPPβ and sAPPα, respectively, as endogenous Aβ17-40 was not detected in IP-MALDI-MS spectra. A comparison of absolute levels of sAPPβ and sAPPα was 1:4.1. Thus, we speculate that the proportion of endogenous APP substrates undergoing processing by the BACE1, α-secretase, and ADAMTS4 is 1:4.1:0.15. These data support our notion that the metalloprotease-mediated proteolytic production pathway for APP669-711 is independent of the Aβ-generating pathway, and is conserved in murine cells.

### Identification of ADAMTS4 as an APP669 site-cleaving enzyme

A comprehensive analysis of various Aβ peptides from GM6001-treated cells suggests that the same metalloprotease is involved in cleaving APP669 and the Aβ 12th positions of Aβ. Recently, it was reported that the Aβ 12th position is cleaved by the secreted metalloprotease ADAMTS4 [23]. ADAMTS4 is identified as aggrecanase-1, which is a major aggrecan-degrading enzyme and is implicated in the development of osteoarthritis [24, 25]. The proteolytic activity of ADAMTS4 is inhibited by TIMP3 [26, 27]. The recognition motif for ADAMTS4 (i.e., E-[AFVLMY]-X[0,1]-[RK]-X[2,3]-[ST]-[VYIFWMLA]) contains Glu at the P1 position and partially corresponds to the (−4th) position in the human Aβ sequence, in addition to the Aβ 3rd and Aβ 11th positions [28]. Of note, the Aβ 3rd position in the murine Aβ sequence does not match with the motif, as the Aβ 5th position is Gly instead of Arg in humans. Walter et al. reported that the overexpression of ADAMTS4 in HEK293 cells expressing APPswe resulted in the overproduction of Aβ4-40 and Aβ12-40 [23]. They also showed that the Aβ peptide starting at the 1st position is also a direct substrate for ADAMTS4. However, they did not investigate the effects of ADAMTS4 on APP669-711 production.

**Fig. 4:**
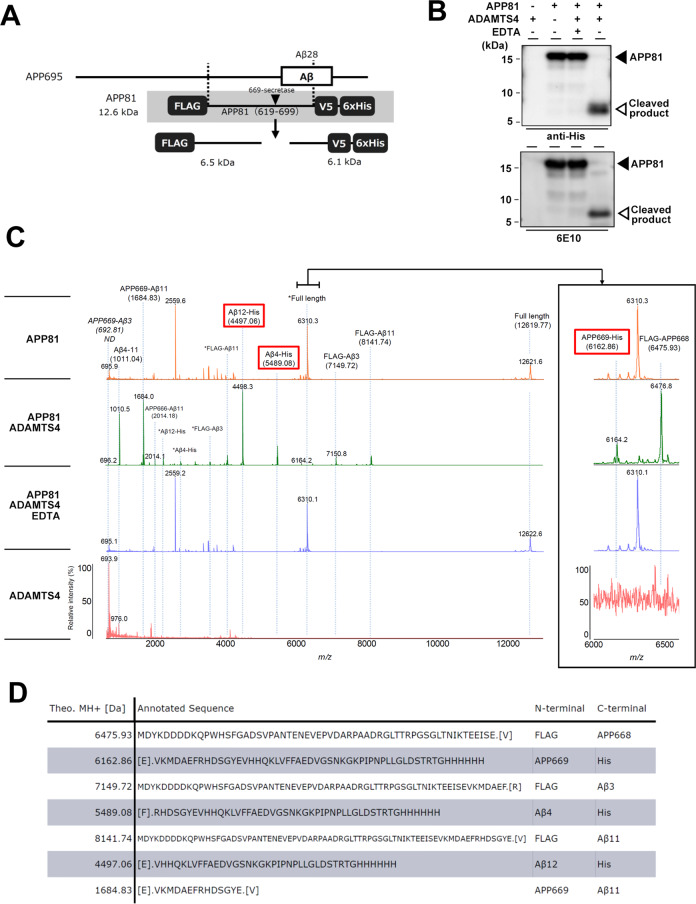
In vitro cleavage assay using recombinant APP81 substrate. **A** Schematic depiction of the recombinant APP81 substrate and the in vitro assay. **B** Immunoblot analysis of APP81 (black triangles) and cleaved products (white triangles) generated by recombinant ADAMTS4. Note that the ADAMTS4-mediated cleavage was inhibited by preincubation with EDTA. **C** MALDI-MS spectrum of the reaction mixture of APP81 and ADAMTS4 (green). Several proteolytic fragments, which were not identified in the substrate only (orange), enzyme only (red), nor EDTA coincubated (blue) samples, were detected. The inset indicates the enlarged spectrum around *m/z* 6000 to 6600. Asterisks indicate double protonated signals. **D** Theoretical molecular weights of peptides specifically identified from the reaction mixture, and their annotated sequences are shown.

To test whether ADAMTS4 can cleave APP directly at the APP669 site, we first performed the in vitro digestion assay using a novel recombinant APP substrate, APP81 (Fig. 4A). APP81 encodes the APP619-699 sequence tagged with N-terminal FLAG and C-terminal V5-His tags. Coincubation of purified APP81 with recombinant ADAMTS4 resulted in the appearance of low-molecular-weight peptides that were recognized by anti-His tag and anti-Aβ (6E10) antibodies (Fig. 4B). This reaction was specifically inhibited by the addition of metal chelator ethylenediaminetetraacetic acid. We then analyzed the proteolyzed peptides by MALDI-TOF-MS (Fig. 4C, D). All N- and C-terminal fragments generated by APP669, Aβ 4th, and Aβ 12th-site cleavages (FLAG-APP668 and APP669-His; FLAG-Aβ3 and Aβ4-His; and FLAG-Aβ11 and Aβ12-His, respectively) were detected. Moreover, a peptide derived from double digestion by APP669 and Aβ 12th-site cleavages (i.e., APP669-Aβ11) was also identified by MS/MS analysis. These data indicate that ADAMTS4 can directly cleave APP at APP669, the 4th and 12th positions.

**Fig. 5:**
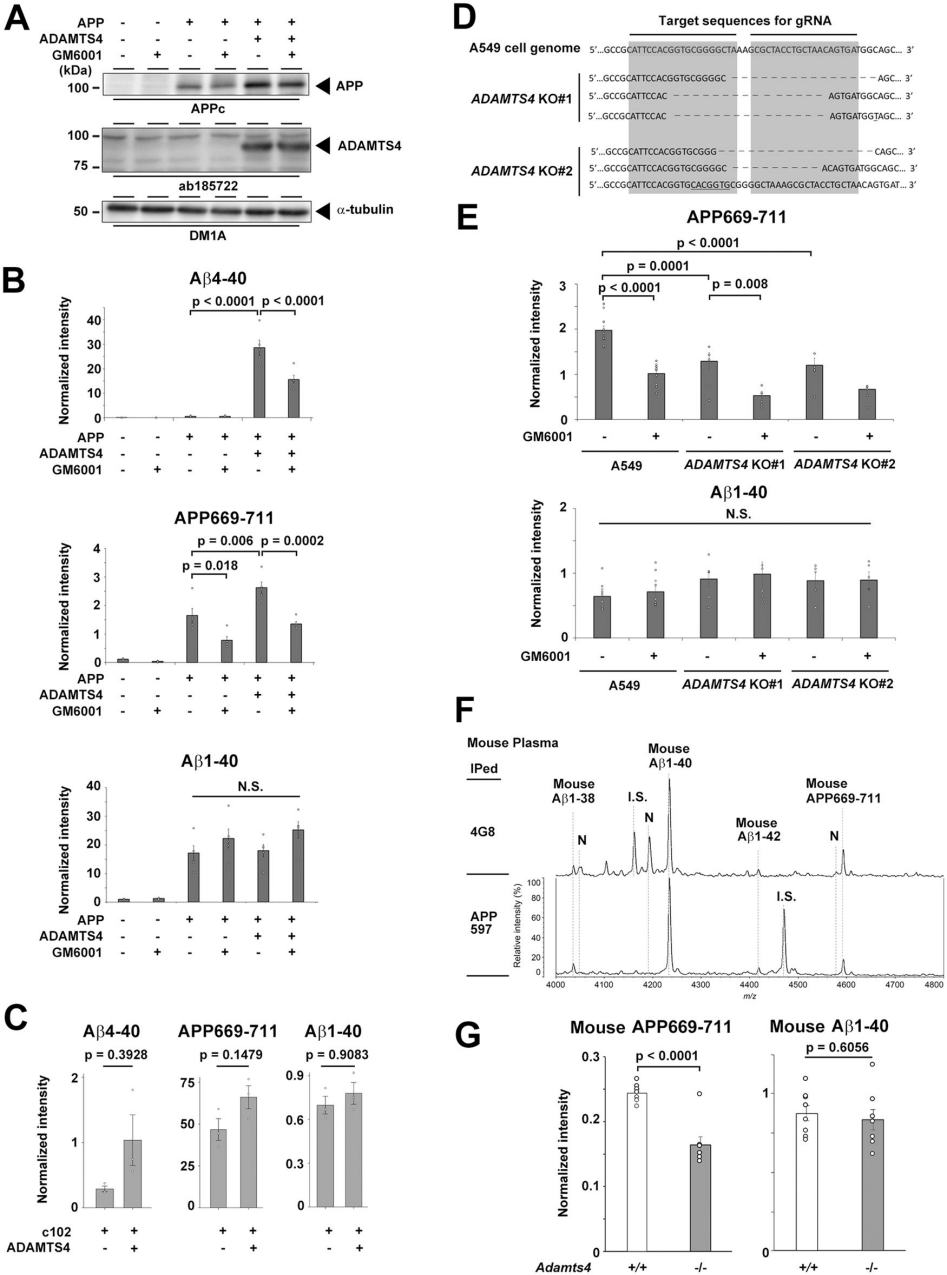
Effects of ADAMTS4 on APP669-711 production. **A** Immunoblot analysis of HEK293A cell lysates expressing APP and ADAMTS4. **B** Levels of Aβ peptides in the conditioned medium of HEK293A cells in **A** (*n* = 4 or 5, mean ± s.e.m. Tukey test. N.S. not significant). **C** Levels of Aβ peptides in the conditioned medium of HEK293A cells expressing c102 and ADAMTS4 (*n* = 3, mean ± s.e.m. Tukey test). **D** Target sequence of the ADAMTS4 locus in A549 cells and genomic sequences of the monoclonal A549 cell lines. **E** Levels of endogenous Aβ peptides in the conditioned medium of A549 cells in **D** (*n* = 4 or 5, mean ± s.e.m. Tukey test. N.S. not significant). **F** IP-MALDI-MS spectrum of the 4G8 or APP597 antibody-precipitated endogenous Aβ peptides in the plasma of wt mice (100 μl for 4G8, 125 μl for APP597). I.S. internal standard (distinct peptides were used for 4G8 and APP597). N non-specific peak. **G** Levels of endogenous APP669-711 and Aβ1-40 in the plasma of wt and *Adamts4* knockout mice (*n* = 7, mean ± s.e.m. Student’s *t* test).

**Fig. 6:**
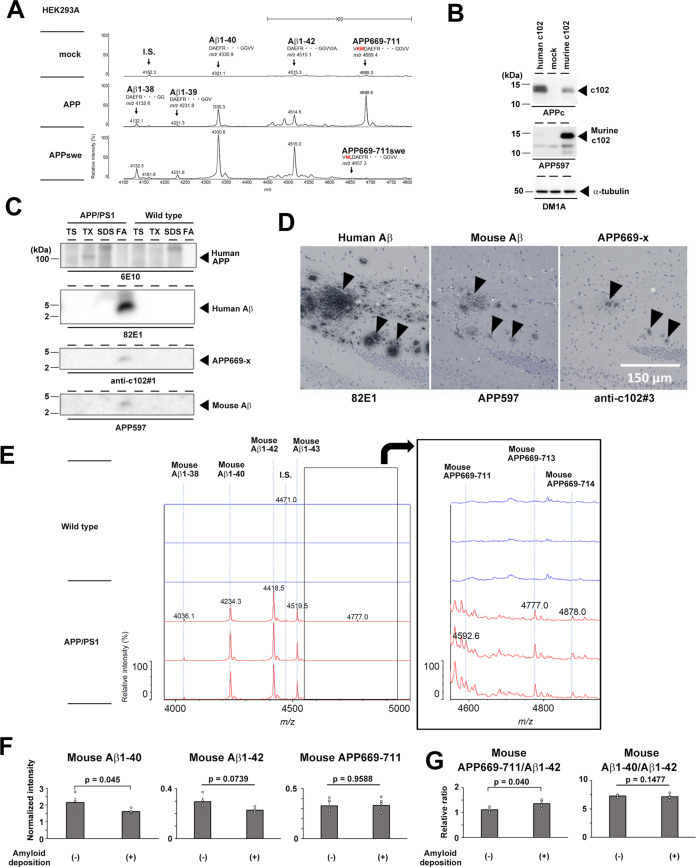
APP669-711 in the Aβ plaque-laden AD model mice. **A** Effects of the Swedish mutation in APP on the production of APP669-711. Levels of Aβ peptides in the conditioned medium of HEK293A cells expressing APPwt and APPswe are shown. **B** The specificity of the APP597 antibody against the murine Aβ sequence was analyzed by immunoblot analysis of HEK293A cells expressing human c102, or murine c102. **C** Immunoblot analysis of brain fractions of aged wt and APP/PS1 mice. TS Tris buffer-soluble fraction, TX 1% Triton-X-soluble fraction, SDS SDS-soluble fraction, FA formic acid-soluble fraction. **D** Immunohistochemical analysis of the brains of aged APP/PS1 mice. Arrowheads indicate Aβ plaques. **E** IP-MALDI-MS analysis using APP597 antibody of FA fraction of APP/PS1 mouse (*n* = 3, 18–22 months old). Wild-type mice (22 months old) were used as a control. Enlarged spectrum in the red box were shown in the inset (black box). **F** Levels of endogenous mouse Aβ peptides in the plasma of young (2 months old, (−)) and aged (23–25 months old, (+)) APP/PS1 mice. The plasma was analyzed by IP-MALDI-MS using the APP597 antibody (*n* = 4 or 5, mean ± s.e.m. Student’s *t* test). **G** Relative ratio of mouse Aβ peptides in the plasma (*n* = 4 or 5, mean ± s.e.m. Student’s *t* test).

Next, we analyzed the conditioned medium of HEK293A cells expressing APPwt and ADAMTS4 (Figs. 5A, B and S1). Consistent with a previous report, the level of Aβ4-40 was significantly increased by ADAMTS4 expression [23]. The level of APP669-711 was also slightly, but significantly upregulated by ADAMTS4, and was reduced by treatment with GM6001 or TIMP3. The production of Aβ1-40 and Aβ1-42 was not altered by ADAMTS4 overexpression, GM6001, or TIMP3 treatment. Furthermore, co-expression of c102 with ADAMTS4 did not increase Aβ4-40 levels (Fig. 5C). Notably, the production of APP669-711 from APPwt expressed in HEK293A cells was unaltered by the addition of the conditioned medium of ADAMTS4-expressing HEK293A cells, suggesting that ADAMTS4 cleavage at the APP669 site occurred on APP in the secretory pathway, rather than the cell surface APP (Fig. S2). We then analyzed the effects of the genetic knockout of ADAMTS4 in A549 cells, which secrete endogenous Aβ and APP669-711. IP-MALDI-MS analysis of the conditioned medium of A549 cells using 6E10 antibody revealed that endogenous Aβ4-40 is hardly produced from this cell line unless ADAMTS4 is overexpressed (Fig. S3). Two monoclonal A549 cell lines that harbor deletion/substitution mutations at the *ADAMTS4* locus by the CRISPR/Cas9 system were selected (Fig. 5D). Both cell lines showed a 30–40% reduction in secreted APP669-711 levels, whereas Aβ production was unaltered (Fig. 5E). Notably, GM6001 treatment further decreased the production of APP669-711 from *ADAMTS4*-knockout A549 cells. Finally, we analyzed the effects of the genetic ablation of *Adamts4* on the level of endogenous APP669-711 in vivo. Human Aβ and mouse Aβ differ by three amino acids (R5/Y10/H13 in human Aβ are G5/H10/R13 in mouse Aβ), and the antibody APP597 was raised against a synthetic peptide encoding murine Aβ (see Fig. 6B, E) [29]. IP-MALD-MS using the 4G8 antibody or the murine Aβ-specific APP597 antibody enabled us to detect endogenous murine Aβ-associated species, such as Aβ1-40, Aβ1-42, and APP669-711 in wt mouse plasma (Fig. 5F). We then applied this method to the plasma samples obtained from *Adamts4*^−/−^ mice [30]. Consistent with the data of *ADAMTS4*-knockout A549 cells, the plasma levels of murine APP669-711 in *Adamts4*^−/−^ mice were 33% lower than those of wt mouse plasma (Fig. 5G). Collectively, these data strongly suggest that ADAMTS4 is involved in the generation of approximately one-third of the APP669-711 in plasma.

### Endogenous murine APP669-711 in the brains of Aβ plaque-laden Alzheimer disease model mice

We and others previously reported that the APP669-711 peptide has the ability to form amyloid fibrils [12, 31]. However, the existence of N-terminally elongated Aβ species in amyloid plaques has not been investigated to date. ADAMTS4 is expressed in the central nervous system [24], and our cell-based assay revealed that neuroblastoma cell secretes APP669-711. Furthermore, APP669-711 was detected in the human CSF [32]. Notably, the intergenic variant located near the *ADAMTS4* gene has been identified as one of the genetic risk factors for sporadic AD by genome-wide meta analysis [33]. To analyze the pathological effects and the diagnostic value of APP669-711 in the brains of Aβ plaque-laden AD model mice, we analyzed aged mice that express human APPswe and mutant *Psen1* (APP/PS1 mice) [34]. Of note, HEK293A cells expressing APPswe produced no APP669-711 carrying the Swedish mutation, suggesting that amino acid substitutions at Aβ (−2nd) and (−1st) sites (i.e., KM670/671NL) abolish the production of APP669-711 from human APP (Fig. 6A). Thus, using the mouse Aβ sequence-specific antibody APP597, we analyzed the effects of the brain deposition of amyloid comprised of human Aβ on the production of APP669-711 from mouse APP, separately from the processing of human APP encoded by the transgene. The specificity of APP597 was confirmed by immunoblot analysis of cell lysate expressing murine c102 (Fig. 6B). For human Aβ, we utilized 82E1 antibody that specifically reacts with N terminus of human Aβ1-x (Fig. 2A). As expected, we detected the deposition of 82E1-positive human Aβ (i.e., human Aβ1-x) in the brain parenchyma and substantial enrichment of human Aβ in the formic acid-soluble fraction. Notably, APP597 reacted with a 4-kDa band corresponding to human Aβ in the formic acid-soluble fraction (Fig. 6C). Furthermore, a similar 4-kDa band was detected by the anti-c102#1 antibody. Consistent with the biochemical analysis, immunohistochemical analysis demonstrated that the plaques in the cortex were positive for APP597 and anti-c102#3 antibodies, suggesting that endogenous murine Aβ peptides including APP669-x peptides formed insoluble aggregates and were deposited in human Aβ plaques. To characterize the deposited endogenous murine Aβ species in the aged APP/PS1 mice, we performed IP-MALDI-MS analysis using APP597. We confirmed that various mouse Aβ peptides, such as Aβ1-38, Aβ1-40, Aβ1-42, and Aβ1-43 were recovered from the formic acid fraction of APP/PS1 mouse brains. In addition, murine APP669-711 (Aβ(-3)-40), APP669-713 (Aβ(-3)-42), and APP669-714 (Aβ(-3)-43) were detected (Fig. 6E). Finally, we analyzed the level of endogenous murine Aβ species in the plasma of aged APP/PS1 mice, which contained a substantial amount of human Aβ, by IP-MALDI-MS using the APP597 antibody. Comparing the peptide levels of various Aβ species in the plasma of young and aged APP/PS1 mice, we found a significant increase in murine APP669-711/murine Aβ_1-42_ ratio in the plasma, as observed in the plasma of humans with Aβ plaques in the brain (Fig. 6F, G). However, the plasma murine Aβ1-40/murine Aβ1-42 ratio was unaltered. These results suggest that, in AD model mice with Aβ plaque development, APP669-711 is produced in the brain and deposited together with human Aβ. In addition, the present data also indicate that the levels of mouse Aβ species in plasma are useful as surrogate biomarkers of amyloid deposition in the brains of AD model mice.

## Discussion

Genetic evidence has highlighted the importance of the proteolytic processing pathway of APP in the pathogenesis of AD [1–3]. In addition to BACE1, which generates the Aβ starting at 1st and 11th positions, several enzymes (e.g., cathepsin B, meprin, and ADAMTS4) were reported as enzymes responsible for producing N-terminally truncated Aβ [35]. In this study, we identified that ADAMTS4 is a key enzyme in the production of the N-terminally elongated Aβ species APP669-711, which is a component of a plasma biomarker of brain amyloid deposition [11, 12]. Our biochemical and cell biological results indicated that APP669-711 is proteolytically produced by the sequential cleavage of APP, similarly to that of Aβ; ADAMTS4 cleaves APP at the APP669 site to generate the C-terminal stub c102, which is a direct substrate for γ-secretase. Notably, the production of APP669-711 increased upon treatment with a BACE1 inhibitor, and the Swedish mutation that enhances BACE1-mediated cleavage abolished the production of APP669-711. Consistent with these results, the Swedish mutation at the (−2nd)(−1st) residues of Aβ (KM to NL) causes a mismatch with the recognition motif for ADAMTS4. Furthermore, APPswe might hinder this processing mechanism both in vitro and in vivo, as suggested previously [35].

Our results also clarified the complex mechanism of APP669-711 production. ADAMTS4 is responsible for 30% to 40% of the secreted APP669-711 in the conditioned medium of cultured cells as well as in mouse plasma in vivo. GM6001 treatment further reduced APP669-711 secretion from *ADAMTS4*-knockout A549 cells. Moreover, the treatment of cells with the protease inhibitor TIMP3 abolished the production of APP669-711. Thus, a TIMP3-sensitive metalloprotease, in addition to ADAMTS4, may also be involved in the APP669-711 production pathway. We are currently analyzing the activities of other proteases that have been reported as TIMP3-sensitive metalloproteases. Another intriguing feature is that although all known APP secretases to date are transmembrane proteins, ADAMTS4 is a secreted protease. This raises the possibility that cells secreting ADAMTS4 are distinct from the substrate-expressing cells. Thus, further cell biological studies are required to demonstrate which cell type(s) is involved, and where the cleavage actually occurs at the subcellular level. However, the generation of APP669-711 was found to be dependent on the γ-secretase activity, indicating that ADAMTS4 cleaves membrane-tethered APP, rather than the secreted Aβ species, in the APP669-711 production pathway. Supporting this hypothesis, almost no Aβ starting at 4th position was secreted from HEK293A cells expressing recombinant c102 or c99, indicating that the C-terminal stubs of APP are not direct substrates for the Aβ 4th site cleavage. Robust ADAMTS4 activity might result in additional cleavage of APP at the Aβ 4th site to generate c96, which is a direct substrate of Aβ4-x. Nevertheless, identification of the APP669-cleaving enzyme(s) and clarifying the precise mechanistic crosstalk in the APP669-711 production pathway is crucial for understanding the biological mechanism of surrogate biomarkers of brain amyloid deposition.

Our study also identified the presence of endogenous murine APP669-711 in mouse plasma. Furthermore, we found an increase in the murine APP669-711/murine Aβ1-42 ratio in the plasma of AD model mice with human Aβ deposition similar to that observed in humans. However, the absolute levels of murine APP669-711, as well as murine Aβ1-42 in plasma were not significantly altered in aged AD model mice, although we observed a reduction in murine Aβ1-40 levels. To date, the origin of the plasma Aβ species remains unclear. Several studies have suggested that a proportion of brain Aβ is effluxed into the blood via the blood-brain barrier, and is then cleared from the liver or kidneys [36–39]. Although ADAMTS4 and APP are expressed in various cells of peripheral tissues, we found that endogenous murine APP669-x as well as Aβ peptides were co-deposited together with human Aβ in the APP/PS1 mouse brains. Thus one possibility is that plasma APP669-711 is originally derived from the brain. Supporting this notion, IP-MALDI-MS analysis revealed the existence of APP669-711 in the human CSF [32]. Pharmacokinetic studies, and understanding the metabolism of APP669-711 as well as murine Aβ are required. Nevertheless, our findings indicate that the murine APP669-711/murine Aβ1-42 ratio in the plasma may be useful for the evaluation of drug efficacy/target engagement in the development of anti-Aβ drugs using AD model mice.

Identification of ADAMTS4 as a responsible enzyme for APP669-site cleavage raises the important possibility of the pathological/diagnostic importance of APP669-711 in humans. Recent genome-wide association studies demonstrated that the intergenic variant *rs4575098* located near the *ADAMTS4* gene is associated with AD [33, 40]. Pathological and biochemical analyses demonstrated that Aβ4-x species, which are generated by ADAMTS4-mediated processing, are deposited in AD brains [23, 41, 42]. Our study also suggested the possibility that APP669-711 is a component of senile plaques. Thus, changes in the expression of *ADAMTS4* might affect the pathological process of AD, possibly by increasing the production of Aβ-related species including APP669-711 and Aβ4-x. Although the average level of plasma APP669-711 in Aβ PET-positive individuals was comparable to that in Aβ-negative individuals, some people showed significantly higher/lower levels of APP669-711 [12]. The balance of ADAMTS4 and TIMP3 levels is crucial in the development of osteoarthritis, in which the overactivation of ADAMTS4 is implicated in its pathogenesis [25]. In addition, some studies showed changes in plasma ADAMTS4 levels in other inflammatory diseases, such as acute coronary syndrome [43]. Although it remains unclear whether plasma ADAMTS4 is directly involved in APP processing in peripheral tissues, these studies suggest the possibility that variations in APP669-711 levels might reflect disease conditions.

In conclusion, we identified ADAMTS4 as an important enzyme involved in APP processing. Further cell biological and genetic analyses are expected to clarify the importance of ADAMTS4, not only in the diagnosis of brain amyloid deposition but also in the pathogenesis of AD.

## Supplementary information

Supplementary Information
