# Supplementary material for: ADAMTS4 is involved in the production of the Alzheimer disease amyloid biomarker APP669-711
Source: Mol Psychiatry. 2023 Feb 1;28(4):1802–12. doi: 10.1038/s41380-023-01946-y (PMC10208957; doi:10.1038/s41380-023-01946-y)
Supplement: Supplementary file 1 — Supplementary Information [file 41380_2023_1946_MOESM1_ESM.pdf]

Supplementary Information

ADAMTS4 is involved in the production of the Alzheimer disease amyloid biomarker APP669-711

Masaya Matsuzaki[1,4], Miyabishara Yokoyama[1,4], Yota Yoshizawa[1,4], Naoki Kaneko[2], Hiroki Naito[2], Honoka Kobayashi[1], Akihito Korenaga[2,], Sadanori Sekiya[2,], Kentaro Ikemura[3], Gabriel Opoku[3], Satoshi Hirohata[3], Shinichi Iwamoto[2,], Koichi Tanaka[2,], Taisuke Tomita[1*]

[1]Laboratory of Neuropathology and Neuroscience, Graduate School of Pharmaceutical Sciences, The University of Tokyo, Tokyo 113-0033, Japan.

[2]Koichi Tanaka Mass Spectrometry Research Laboratory, Shimadzu Corporation, Kyoto 604-8511, Japan.

[3] Department of Medical Technology, Graduate School of Health Sciences, Okayama University, 2-5-1, Shikata-cho, Okayama, Japan.

[4]These authors contributed equally to this work.

Corresponding author

*To whom correspondence should be addressed: Taisuke Tomita, Ph.D.

Laboratory of Neuropathology and Neuroscience, Graduate School of Pharmaceutical Sciences, The University of Tokyo

7-3-1 Hongo, Bunkyo-ku, Tokyo 113-0033, Japan

E-mail: taisuke@mol.f.u-tokyo.ac.jp

ORCID ID: 0000-0002-0075-5943

Running title

ADAMTS4 cleaves APP at the APP669 site

Supplementary Materials and methods

Animals

All experiments using animals were performed according to the guidelines of the Institutional Animal Care Committee of Graduate School of Pharmaceutical Sciences, The University of Tokyo (protocol no. P29-30 and P30-3), and Graduate School of Health Sciences, Okayama University (study approval no. OKU2022-413). APP/PS1 mice (B6.Cg-Tg(APPswe, PSEN1dE9)85Dbo/Mmjax, Jackson Laboratory, JAX mouse #005864) express a chimeric mouse/human APP with the Swedish mutation (K670M/K671L), and the human PSEN1 gene with an exon 9 deletion, expressed under the control of the murine prion promoter. *Adamts4*$^{-/-}$ mice (B6.129P2-Adamts4tm1Dgen/J, Jackson Laboratory, JAX mouse #005770) harbor a bacterial *lacZ* gene that was inserted into the locus of the *Adamts4* gene, such that the endogenous *Adamts4* gene promoter drives the expression of β-galactosidase. For serological analyses, 17-week-old *Adamts4*$^{-/-}$ littermate male (n = 4) and female (n = 4) mice were used. Animals were anesthetized with an intraperitoneal injection of pentobarbital sodium (50 mg/kg), and venous blood was collected to obtain plasma. Then, the mice were perfused with PBS, and tissues were collected and snap-frozen in liquid nitrogen and stored at −80 °C until use.

Antibodies and chemicals

The following primary antibodies were used in this study: anti-human Aβ 82E1 (1:1,000; IBL #10323), anti-human Aβ 6E10 (1:1,000; BioLegend #SIG-39300), anti-Aβ 4G8 (1:1,000; BioLegend #SIG-39200), anti-BACE1 BACE1c (1:1,000; IBL #18711), anti-APP APPc (1:1,000;

IBL #18961), anti-sAPPβ-Wild Type (1:1,000; IBL #18957), anti-Mouse/Rat APP597 (1:1,000; IBL #28055), anti-APP A4 (1:100; Merck #22C11), anti-ADAMTS4 (1:1,000; Abcam, ab185722), anti-c-myc (1:1,000; Cell Signaling Technology, #9B11) and anti-α-tubulin (1:5,000; Sigma-Aldrich DM1A). For the generation of N-terminal end-specific anti-c102 antibodies, a synthetic antigen peptide encoding VKMDAEF-amino linker-C conjugated with keyhole limpet hemocyanin was injected into a rabbit, and serum was purified by antigen peptides immobilized to SulfoLink Coupling Resin (Thermo Fisher Scientific). The following chemicals were used in this study: EDTA (DOJINDO, the stock solution was dissolved in distilled water to a final concentration of 1 mM), complete protease inhibitor cocktail (Roche Applied Science), complete protease inhibitor cocktail EDTA free (Roche Applied Science), GM6001 (Enzo Life Science #BML-EI300, the stock solution was dissolved in DMSO to a final concentration of 25 μM), recombinant human TIMP3 (R&D systems # 973-TM, the stock solution was dissolved in distilled water to a final concentration of 100 μg/mL). The ADAM10/17 inhibitor INCB3619 [1], β-secretase inhibitor MBSI [2], and γ-secretase inhibitor DAPT [3] (the stock solution was dissolved in DMSO to a final concentration of 10 mM) were kindly provided by Drs. Tohru Fukuyama and Satoshi Yokoshima (Nagoya University). For the detection of murine sAPPβ and sAPPα in the conditioned medium of N2a cells, we utilized ELISA kits specifically detecting sAPPβ (IBL #27416) and sAPPα (IBL #27419), respectively.

Cell culture and transfection

CCF-STTG1 (#90021502) and BE(2)-C (#95011817) cells were purchased from the European Collection of Authenticated Cell Cultures. The A549 cell line (JCRB0076) was

purchased from JCRB Cell Bank, Japan. H4 (HTB-148) cells were purchased from American Type Culture Collection. Neuro2a [4] and HEK293A [1] cells were kindly provided by Drs. Kei Maruyama (Saitama Medical University) and Hidenori Ichijo (The University of Tokyo), respectively. Cells were cultured in Dulbecco's modified Eagle medium (DMEM) with high glucose (Wako Chemicals) supplemented with 10% heat-inactivated fetal bovine serum (FBS; Hyclone), 50 units/mL penicillin (Invitrogen), and 50 mg/mL streptomycin (Invitrogen) at 37 °C under humidified air containing 5% CO_2. The possibility of mycoplasma contamination was routinely checked by DAPI staining and PCR analysis. Human *ADAMTS4* and *TIMP3* cDNAs were amplified from an in-house human cDNA library derived from total RNAs of BE(2)-C cells using KOD plus neo DNA polymerase (Toyobo), and the primers listed in Appendix Table Sx. For the overexpression experiment in mammalian cells, *ADAMTS4* and *TIMP3* cDNAs were inserted into the pcDNA3.1/Hygro(+) vector (Invitrogen) by HiFi Assembly (New England BioLabs). Expression vectors for APPwt, APPswe, and c99 were described previously [4–9]. The c102 expression vector was generated by the insertion of three amino acids (i.e., VKM) into the c99 sequence using PCR-mediated mutagenesis with the primers shown in Appendix Table S2. For the generation of CRISPR-knockout cells, we Cas9 nickase (D10A) was used, which requires two adjacent guide RNAs (gRNA) for cleaving target regions [10–12]. cDNAs encoding gRNA sequences (Appendix Table S3) were inserted into pX335-U6-Chimeric_BB-CBh-hSpCas9n(D10A) (a gift from Dr. Feng Zhang, Addgene plasmid #42335; http://n2t.net/addgene:42335; RRID: Addgene_42335) and pBabe Puro U6 BbsI (kindly provided by Dr. Dario Alessi [University of Dundee]). Transfection into HEK293A and A549 cells using polyethylenimine (Polysciences) and LipofectAMINE LTX (Invitrogen), respectively, were

described previously [10, 11]. To analyze the effect of extracellular ADAMTS4, HEK293A cells overexpressing APPwt were incubated with the conditioned medium from HEK293A cells after 48h of transfection by ADAMTS4. Then the conditioned medium was subjected to IP-MALDI-MS analysis.

Generation of CRISPR knockout cells

After transfection, media were replaced with fresh media containing puromycin at 2 μg/mL. For establishing monoclonal cells, cells after puromycin selection were seeded onto 96-well plates coated with 0.1% (w/v) gelatin (Fujifilm Wako; #190-15 805), and cultured in DMEM containing 30% (v/v) FBS. After reaching approximately 80% confluency, individual clones were transferred to 6-well plates. The genome from monoclonal cells was obtained by NucreoSpin Tissue (MACHEREY-NAGEL), and amplified by Sanger sequencing. PCR products were inserted into p3×FLAG-CMV-10 vector (Sigma-Aldrich) by HiFi assembly, and then transformed into *E. coli* DH5α cells. Plasmids were isolated using a miniprep kit (Nippon Genetics) and sequenced to confirm frameshift/point mutations. A549 cells have 5.8 ± 1.40 copies of chromosome 1 per karyotype [13], which is where the *ADAMTS4* gene is located. Thus, we chose a monoclonal cell line that harbors three mutations within the target genomic sequence.

Preparation of samples for biochemical analyses

For the preparation of total cell lysates, cells were lysed in 2% SDS, briefly sonicated, and then incubated at 37 °C for 30 min with 1% 2-mercaptoethanol. For the preparation of conditioned

medium, the collected medium was spun down at 15,000 × *g* for 10 min at 4 °C, and stored at −80 °C until use. Mouse brains were fractionated as previously described [14, 15]. Briefly, brains were homogenized in a 10× volume of Tris buffer (50 mM Tris HCl [pH 7.6], 150 mM NaCl, Complete protease inhibitor cocktail, and PhosSTOP phosphatase inhibitor cocktail) with 25 strokes using a mechanical homogenizer, and centrifuged at 200,000 × *g* for 20 min at 4 °C. The resultant supernatant was collected as the brain Tris buffer-soluble fraction. After the addition of the same amount of 2% Triton X-100/Tris buffer, the pellet was homogenized on ice and centrifuged at 200,000 × *g* for 20 min at 4 °C. The resultant supernatant was collected as the brain Triton X-fraction. Then, the same amount of 2% SDS containing Tris buffer was added to the pellet. After homogenization at room temperature, the pellet was incubated for 2 hours at 37 °C and centrifuged at 200,000 × *g* for 20 min at 20 °C. The resultant supernatant was collected as the brain SDS fraction. Finally, the pellet was sonicated (BRANSON, output: 2; duty cycle: 90; 15 sec) with 500 μL of 70% formic acid solution (Wako) Samples were centrifuged at 200,000 × *g* for 20 min at 4 °C, and the resultant supernatant was freeze-dried for 2 hours (Thermo Scientific, Savant RVT5105). The pellet was dissolved in the same volume of DMSO (Wako) as the brain weight, and stored at −80 °C until use. Protein concentrations of the fractions were measured by the BCA protein assay (Pierce). To detect plasma Aβ, mouse blood was collected from the right ventricle or the facial vein near the jawbone of the mouse, and mixed with EDTA (final concentration: 0.15%). Plasma were obtained by centrifugation at 3,500 rpm for 5 min at 4 °C, and stored at −80 °C until use.

Immunoblotting

Immunoblotting analyses were performed as previously described [4]. Briefly, samples were dissolved in Laemmli sample buffer (final concentration of 1 M Tris-HCl [pH 6.8], 20% SDS, 30% glycerol, 1% Brilliant Green [Wako], 1% CBB-G250 [Nacalai Tesque]) and separated by SDS-PAGE. Gels were transferred to a polyvinylidene difluoride membrane (Millipore). The membranes were incubated in 5% skim milk, treated with primary antibodies diluted in Immuno-enhancer Reagent A (Fujifilm), and then probed with horseradish peroxidase-conjugated secondary antibodies (GE Healthcare and Jackson Immunoresearch) in Immuno-enhancer Reagent B (Fujifilm). Chemiluminescent signals were developed using the ImmunoStar detection kit (Wako), and acquired using ImageQuant LAS 4000 (GE Healthcare). Band intensities were measured using ImageJ software (NIH).

Immunoprecipitation (IP)

Levels of various Aβ species in the samples were measured using the previously described IP-MALDI-MS with some modifications. Briefly, samples were mixed with an equal volume of Tris buffer containing stable-isotope-labeled (SIL) Aβs as internal standard peptides, 0.2% w/v n-dodecyl-β-D-maltoside (DDM) and 0.2% w/v n-nonyl-β-D-thiomaltoside. SIL-human Aβ1-38 and SIL-human Aβ1-15 were used for human Aβ analysis, and SIL-murine Aβ1-42 for murine Aβ analysis. The various Aβ peptide species and internal standards were immunoprecipitated by incubating the samples with antibody beads, which were prepared by coupling anti-Aβ antibodies directly to Dynabeads M-270 Epoxy (Thermo Fisher Scientific). Anti-Mouse/Rat APP597 or anti-

Aβ 4G8 was used for the IP of murine Aβ, and anti-human Aβ 6E10 for the IP of human Aβ. After incubation for 1 hour, the bound peptides were washed and eluted with glycine buffer (pH 2.8) containing 0.1% (w/v) DDM. After the pH was adjusted to 7.4, the IP was repeated once and the bound peptides were eluted with 70% acetonitrile containing 5 mM HCl and 0.1 mM L-methionine. The eluted peptides were applied to 4 wells of a 900 µm µFocus MALDI plate^{TM} (Hudson Surface Technology, Inc.) which was spotted with α-cyano-4-hydroxycinnamic acid (CHCA) and methanediphosphonic acid (MDPNA) in advance.

In vitro cleavage assay

For purification of the recombinant substrate, cDNA encoding APP81 (corresponding to Q619-K699 of the APP770 sequence with the start methionine) was amplified from human APP cDNA, and ligated into the pET-DEST42 vector (Invitrogen) using HiFi Assembly Master Mix (New England BioLabs) and primers (Appendix Table S1) to generate an expression vector for the APP81-V5/His protein. The FLAG tag sequence at the N terminus was then inserted by PCR-mediated mutagenesis with the primers shown in Appendix Table S2. The plasmids were transformed into *E. coli* BL21(DE3) (Novagen), and overexpression was induced by 0.1 mM Isopropyl β-D-1-thiogalactopyranoside. After sonication of the cell pellet in lysis buffer (50 mM Tris-HCl [pH 7.5], 1 mg/mL lysozyme, complete protease inhibitor cocktail, EDTA free), the overexpressed proteins bound to QIAGEN Ni-NTA agarose were purified using elution buffer (50 mM Tris-HCl [pH 7.5], 100 mM imidazole). After dialysis to TS buffer (50 mM Tris-HCl [pH 7.5], 150 mL NaCl), the aliquoted purified proteins were stored at –80 °C until use. Recombinant human ADAMTS4 protein (#4307-AD-020) was purchased from R&D systems. The same volume

(80 μL) of 17 μM APP81 and 100 nM ADAMTS4 protein (diluted in assay buffer [50 mM HEPES, 50 mM NaCl, 1 mM CaCl2, 0.05% Brij-35, pH 7.5]) were mixed and incubated at 37 °C for the indicated hours. For immunoblotting, the reaction was halted by the addition of 2× Laemmli sample buffer. For MALDI-TOF MS, the mixture was stored at −80 °C until use.

Solid-phase extraction (SPE)

Mixtures obtained from the *in vitro* cleavage assay were diluted with 0.1% (v/v) trifluoroacetic acid (TFA), and applied to a ZipTip μ-C_{18} column (Merck) for desalting. The ZipTip was conditioned with 40% (v/v) acetonitrile containing 0.1% (v/v) TFA, and equibrated with 0.1% (v/v) TFA. The mixture containing the peptides was then loaded onto the ZipTip and washed with 0.1% (v/v) TFA. The peptides were eluted from the ZipTip with 40% (v/v) acetonitrile containing 0.1% (v/v) TFA, and mixed with CHCA solution (0.5 mg/mL [w/v] CHCA, 0.2% [w/v] MDPNA, 0.05% [v/v] TFA) or 2,5-dihydroxybenzoic acid (DHB) solution (2.5 mg/mL [w/v] DHB, 0.2% [w/v] MDPNA, 0.05% [v/v] TFA) on a μFocus MALDI plate™.

MALDI-TOF MS

Mass spectra were acquired using a MALDI-linear TOF mass spectrometer (AXIMA Performance, Shimadzu/KRATOS) in the positive ion mode. MS/MS analysis for the identification of the proteolyzed peptides was performed using a MALDI-QIT reflectron TOF mass spectrometer (AXIMA Resonance, Shimadzu/KRATOS) in the positive ion mode, and MS/MS product ions were generated by collision-induced dissociation with argon gas. The *m/z*

reported in the linear TOF and the QIT-reflectron TOF represent the average and monoisotopic peak of the protonated signal $[M + H]^+$, respectively. The *m/z* value was calibrated with human angiotensin II, human adrenocorticotropic hormone (ACTH) fragment 18–39, bovine insulin oxidized beta-chain, and bovine insulin.

In IP-MALDI-MS, the peak intensities were extracted using Mass++ software ver. 2.4.1 (Shimadzu) with a peptide mass tolerance of 4.0 Da. The limit of detection was established at a signal-to-noise ratio of 3:1. One assay produced four mass spectra, and the normalized intensities of the various Aβ peptide variants were obtained by averaging the peak intensities normalized with the standard peptides. The normalized intensities were used as the levels of the Aβ peptide variants.

In MS/MS analysis, the monoisotopic mass lists of the product ions were generated from the raw MS/MS spectra by Mascot Distiller (Version 2.7.1, Matrix Science) as follows: a maximum iteration of 500, a correlation threshold of 0.65, and a minimum peak half-width of 0.01. The generated peak lists were searched against sequences of FLAG-APP81-V5/His and recombinant human ADAMTS4 using the Mascot algorithm (version 2.8.0.1, Matrix Science). The Mascot search parameters were as follows: no enzyme, no missed cleavage, and tolerances of 0.3 Da and 0.3 Da for the precursor and product ions, respectively. Variable modification of oxidation (M) was only used for the oxidized peptide.

Immunohistochemistry

Immunohistochemical analyses were performed as previously described [15–17]. Mice were anesthetized and perfused with 10 mL PBS, sacrificed by decapitation, and their brains were

fixed by soaking them in PBS containing 4% paraformaldehyde overnight. The brains were then rinsed in PBS, and then serially dehydrated in 70%, 80%, 90%, and 99% ethanol (Wako), and then the ethanol was replaced by incubation in xylene (Wako) twice, and then further replaced by incubation in paraffin (Wakko) three times. Paraffin sections (4 μm) were prepared using a microtome (Microedge Instruments, Inc.). Paraffin sections were soaked in xylene three times for 5 min, in ethanol (99%, 90%, 80%, and 70%) for 1 min each, and then autoclaved with 0.1 M citrate buffer solution (pH 6.0) at 121 °C for 10 min, for antigen retrieval. The sections were blocked with PBS containing 10% cow serum for 30 min, and then incubated with primary antibodies overnight, followed by incubation with biotin-conjugated secondary antibodies for 2 hours. The bound antibodies were visualized by ABC elite (Vector) and ImmPACT SG (Vector). After counterstaining with hematoxylin, the samples were mounted with HSR Mounting Medium (Sysmex), and viewed using an EVOS FL Auto2 microscope (Thermo Fisher Scientific).

Statistical analysis

All samples were analyzed in a randomized manner. For quantitative immunoblot analysis, and immunofluorescence, in cells and mice, the Student *t*-test was used for comparisons between two-group data, and the Tukey test was used for multiple group comparisons. Statistical analyses were performed by KyPlot or Excel software. A *p*-value of less than 0.05 was considered to indicate a statistically significant difference between groups

Supplementary References

1. Suzuki K, Hayashi Y, Nakahara S, Kumazaki H, Prox J, Horiuchi K, et al. Activity-Dependent Proteolytic Cleavage of Neuroligin-1. Neuron. 2012;76.

2. Miyagawa T, Ebinuma I, Morohashi Y, Hori Y, Chang MY, Hattori H, et al. BIN1 regulates BACE1 intracellular trafficking and amyloid-β production. Hum Mol Genet. 2016;25.

3. Kan T, Tominari Y, Morohashi Y, Natsugari H, Tomita T, Iwatsubo T, et al. Solid-phase synthesis of photoaffinity probes: Highly efficient incorporation of biotin-tag and cross-linking groups. Chem Commun. 2003;9.

4. Tomita T, Maruyama K, Saido TCTC, Kume H, Shinozaki K, Tokuhiro S, et al. The presenilin 2 mutation (N141I) linked to familial Alzheimer disease (Volga German families) increases the secretion of amyloid β protein ending at the 42nd (or 43rd) residue. Proc Natl Acad Sci U S A. 1997;94:2025–2030.

5. Iwata H, Tomita T, Maruyama K, Iwatsubo T. Subcellular Compartment and Molecular Subdomain of β-Amyloid Precursor Protein Relevant to the Aβ42-promoting Effects of Alzheimer Mutant Presenilin 2. J Biol Chem. 2001;276.

6. Watanabe N, Takagi S, Tominaga A, Tomita T, Iwatsubo T. Functional analysis of the transmembrane domains of presenilin 1: Participation of transmembrane domains 2 and 6 in the formation of initial substrate-binding site of γ-secretase. J Biol Chem. 2010;285.

7. Imamura Y, Watanabe N, Umezawa N, Iwatsubo T, Kato N, Tomita T, et al. Inhibition of γ-secretase activity by helical β-peptide foldamers. J Am Chem Soc. 2009;131.

8. Baba M, Nakajo S, Tu P-H, Tomita T, Nakaya K, Lee VM-Y, et al. Aggregation of α-synuclein in Lewy bodies of sporadic Parkinson's disease and dementia with Lewy bodies. Am J Pathol. 1998;152.

9. Tomita T, Tokuhiro S, Hashimoto T, Aiba K, Saldo TC, Maruyama K, et al. Molecular dissection of domains in mutant presenilin 2 that mediate overproduction of amyloidogenic forms of amyloid β peptides: Inability of truncated forms of PS2 with familial Alzheimer's disease mutation to increase secretion of Aβ42. J Biol Chem. 1998;273.

10. Araki M, Ito K, Takatori S, Ito G, Tomita T. BORCS6 is involved in the enlargement of lung lamellar bodies in Lrrk2 knockout mice. Hum Mol Genet. 2021;30:1618–1631.

11. Chiu YW, Hori Y, Ebinuma I, Sato H, Hara N, Ikeuchi T, et al. Identification of calcium and integrin-binding protein 1 as a novel regulator of production of amyloid β peptide using CRISPR/Cas9-based screening system. FASEB J. 2020;34:7661–7674.

12. Gopalappa R, Suresh B, Ramakrishna S, Kim HH. Paired D10A Cas9 nickases are sometimes more efficient than individual nucleases for gene disruption. Nucleic Acids Res. 2018;46.

13. Isaka T, Nestor AL, Takada T, Allison DC. Chromosomal variations within aneuploid cancer lines. J Histochem Cytochem. 2003;51:1343–1353.

14. Kikuchi K, Tatebe T, Sudo Y, Yokoyama M, Kidana K, Chiu YW, et al. GPR120 signaling controls amyloid-b degrading activity of matrix metalloproteinases. J Neurosci. 2021;41:6173–6185.

15. Kidana K, Tatebe T, Ito K, Hara N, Kakita A, Saito T, et al. Loss of kallikrein-related peptidase 7 exacerbates amyloid pathology in Alzheimer's disease model mice. EMBO Mol Med. 2018;10.

16. Hori Y, Takeda S, Cho H, Wegmann S, Shoup TM, Takahashi K, et al. A Food and Drug Administration-approved asthma therapeutic agent impacts amyloid β in the brain in a transgenic model of Alzheimer disease. J Biol Chem. 2015;290:1966–1978.

17. Kanatsu K, Hori Y, Takatori S, Watanabe T, Iwatsubo T, Tomita T. Partial loss of CALM function reduces Aβ42 production and amyloid deposition in vivo. Hum Mol Genet. 2016;25:3988–3997.

Supplementary Figures

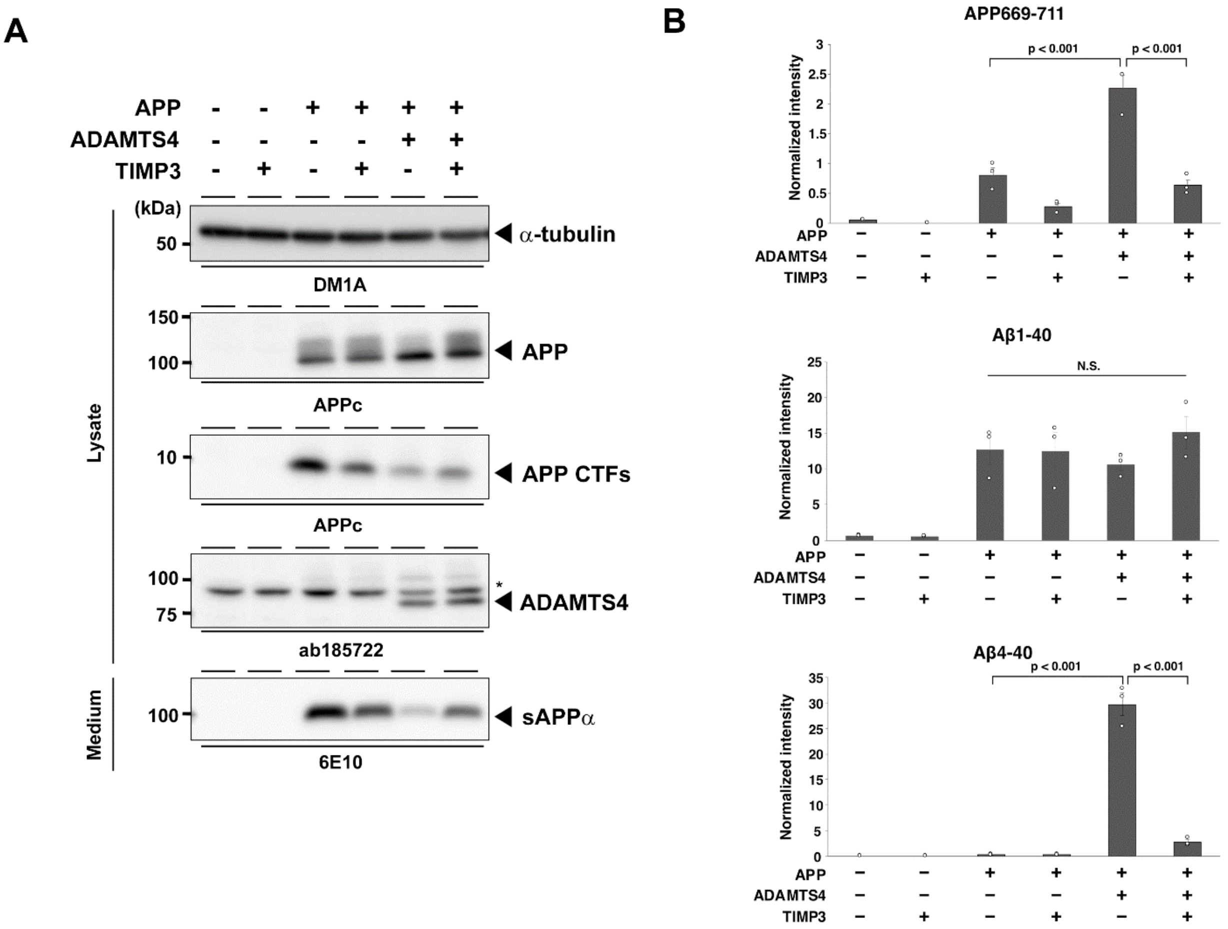

Figure S1. Effect of TIMP3 on APP processing in HEK293A cells.

(A) Immunoblot analysis of HEK293A cells expressing APP, ADAMTS4, and TIMP3. Astersisk indicates non-specific band. (B) Levels of APP669-711 (upper), Aβ1-40 (middle) and Aβ4-40 (lower) secreted from HEK293A cells expressing mock, APP and APP/ADAMTS4. Note that TIMP3 treatment was specifically inhibited the production of APP669-711 in a similar fashion to that observed in N2a cells.

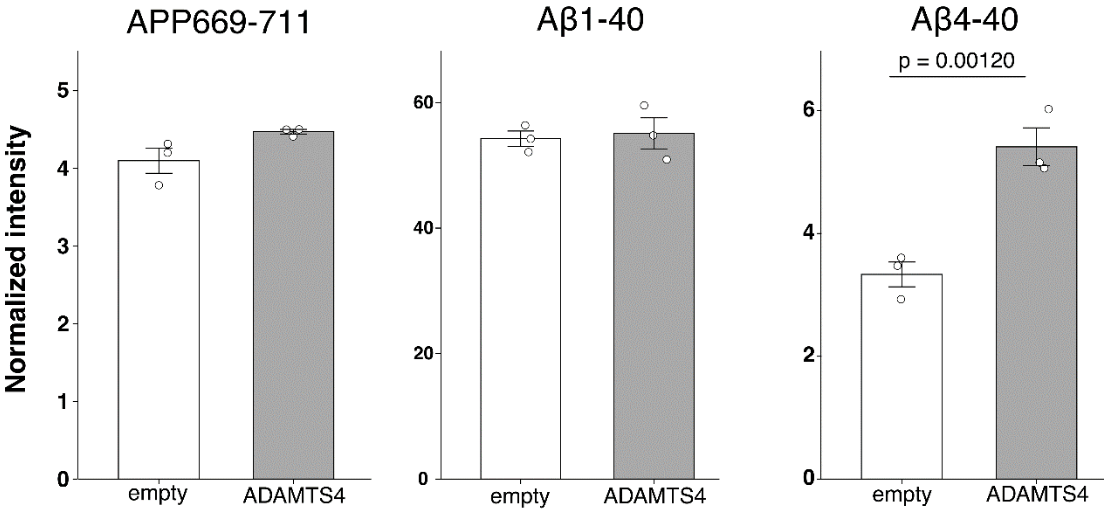

Figure S2. Effect of extracellular ADAMTS4 on APP processing in HEK293A cells.

Levels of Abeta related peptides secreted from APP-expressing HEK293A cells treated with the conditioned medium of mock or ADAMTS4-expressing HEK293A cells. Note that Aβ4-40 level was increased by the ADAMTS4-containing media (n = 3, mean ± s.e.m., Tukey test).

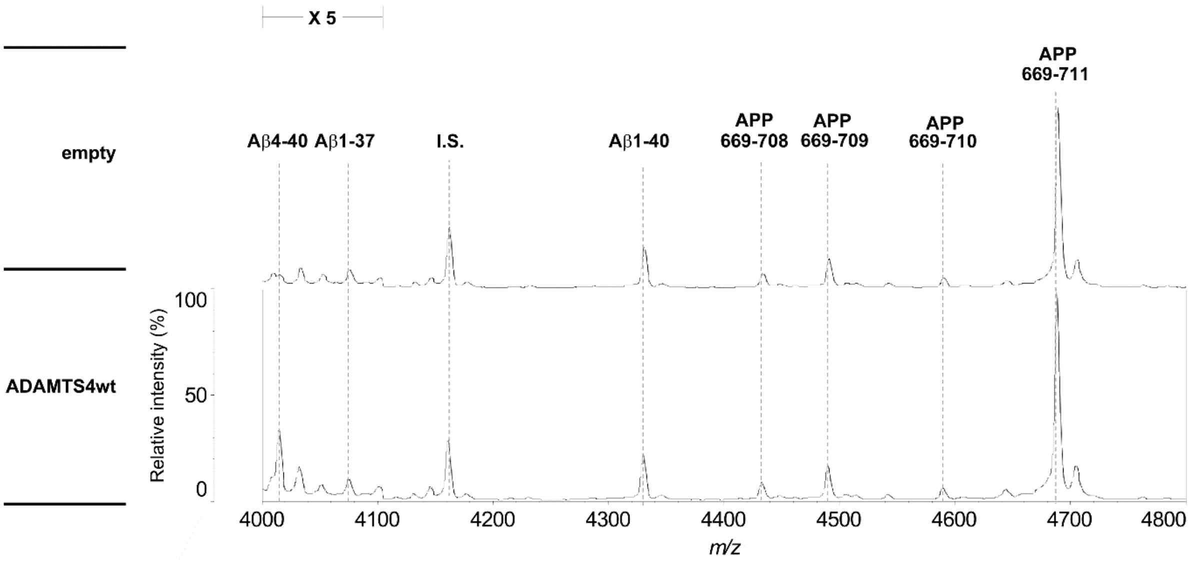

Figure S3. Secreted Aβ peptides in the conditioned medium of A549 cells.

IP-MALDI-MS spectrum of the 6E10 antibody-precipated Aβ peptide variants in the conditioned medium of A549 cells transfected with an empty vector or ADAMTS4wt-encoded vector. Note that Aβ4-40 was detected only in the A549 cells expressing ADAMTS4wt. I.S., internal standard.

Supplementary Table

Table S1 Primer sets used for cDNA cloning

Gene	Sequence (5' to 3')
ADAMTS4	
Forward	GGAGACCCAAGCTGGACCATGTCCCAGACAGGCTCGC
Reverse	ACCAAGCTTAAGTTTAAACGCTTATTTCCTGCCCGCCCAGG
TIMP3	
Forward	TTCTGCAGATACCATGACCCCTTGGCTCGGGC
Reverse-1	GCTTCTGCTCGGGGTCTGTGGCATTGATGATG -
Reverse-2 for myc tag	ACTGTGCTGGATTCACAGGTCCTCCTCTGAGATCAGCTTCTGCTC
APP81	
Forward-1 for insert A	ATGCAGCCGTGGCATTCTTTTGG
Reverse-1 for insert A	CTTTGTTTGAACCCACATCTTCTGCAAAGAAC
Forward-2 for insert B	CTTTAAGAAGGAATATCATGCAGCCGTGGCATTC
Reverse-2 for insert B	AGGGATAGGCTTACCTTTGTTTGAACCCAC
Forward-3 for plasmid C	GGTAAGCCTATCCCTAACCCTCTCC

Reverse-3 for plasmid C	GATATTCCTTCTTAAAGTTAAACAAAATTATTTCTAGAGGGGA

Table S2 Primer sets used for mutagenesis

Gene	Sequence (5’ to 3’)
c102	
Forward	GCTCGGGCGGATGCAGTGAAGATGGATGCAGAATTCCGACATGACTCAG
Reverse	CGAGCCCGCCTACGTCACTTCTACCTACGTCTTAAGGCTGTACTGAGTC
Murine c102	
R676G Forward	GATGCAGAATTCGGACATGACTCAG
R676G Reverse	CTGAGTCATGTCCGAATTCTGCATC
Y681F Forward	GACTCAGGATTTGAAGTTCATCATC
Y681F Reverse	GATGATGAACTTCAAATCCTGAGTC
H684R Forward	GATTTGAAGTTCGCCATCAAAAATTG
H684R Reverse	CAATTTTTGATGGCGAACTTCAAATC
FLAG tag for APP81	
Forward	ATGGACTACAAAGACGATGACGACAAGCAGCCGTGGCATTCTTTT
Reverse	CTTGTCGTCATCGTCTTTGTAGTCCATGATATTCCTTCTTAAAG

Table S3 guide RNA sequences

Gene	Sequence (5' to 3')
ADAMTS4	
for px335	GCGCTACCTGCTAACAGTGA
for pBabe Puro U6 BbsI	CATTCCACGGTGCGGGGCTA
